# The Role of Exopolysaccharides in Direct Interspecies Electron Transfer

**DOI:** 10.3389/fmicb.2022.927246

**Published:** 2022-06-16

**Authors:** Zheng Zhuang, Xue Xia, Guiqin Yang, Li Zhuang

**Affiliations:** Guangdong Key Laboratory of Environmental Pollution and Health, School of Environment, Jinan University, Guangzhou, China

**Keywords:** exopolysaccharides, direct interspecies electron transfer, aggregates, pili, *c*-type cytochromes, conductive material

## Abstract

Direct interspecies electron transfer (DIET) is an effective mechanism for microbial species to exchange electrons cooperatively during syntrophic metabolism. It is generally accepted that DIET is mainly mediated by electrically conductive pili and outer surface *c*-type cytochromes (*c*-Cyts). However, as an extracellular matrix is ubiquitous and abundant on the surface of microorganisms, the effect and mechanism of exopolysaccharides on DIET are still unclear. This study constructed a co-culture of exopolysaccharides-deficient *Geobacter sulfurreducens* with *Geobacter metallireducens* to explore the role of exopolysaccharides in DIET. Results revealed that the deficiency of exopolysaccharides extended the metabolic period of the co-culture by 44.4% and changed the proportions of each species in the co-culture. The exopolysaccharides-deficient co-culture failed to form large, tight spherical aggregates and the expression of *c*-Cyts and pili was decreased. The addition of magnetite and granular activated carbon (GAC), respectively, might compensate for the functions of *c*-Cyts and pili in the first generation of co-culture, but the stimulatory effect on the metabolic stable period co-culture was fairly limited. These findings demonstrate that non-conductive exopolysaccharides are an important component of DIET aggregates and an extracellular matrix for DIET-required *c*-Cyts.

## Introduction

Microorganisms in nature generally do not exist alone, but form complex and diverse relationships with surrounding microorganisms (Ratzke et al., 2020). Syntrophy enables a microbial community to survive with minimal energy through microbial metabolic cooperation, which is conducive to breaking the thermodynamic limit of the substrate metabolism (Dolfing et al., 2008; Morris et al., 2013). Interspecies electron transfer (IET) is a special mechanism of syntrophic microorganisms, relying on electron transfer between dependent microbial partners (Shrestha and Rotaru, 2014; Kouzuma et al., 2015). The earliest IET was proposed as mediated IET (MIET) with hydrogen or formic acid as electron carriers, in which electron transfer between syntrophic species occurs *via* the diffusion of electron carriers, thus direct contact is not required between species (Stams and Plugge, 2009). Direct IET (DIET), a newly discovered alternative to MIET, achieves IET through an electrical connection between syntrophic cells, which has the potential for faster electron transfer and less energy loss than electron carrier diffusion (Summers et al., 2010; Wang et al., 2016). Relevant studies show that DIET not only promotes energy generation in an anaerobic digester, but also has an important function in the biogeochemical cycle of many essential elements (Morita et al., 2011; Wegener et al., 2015; Ha et al., 2017).

The co-culture of *Geobacter metallireducens* (*G. metallireducens*) and *Geobacter sulfurreducens* (*G. sulfurreducens*) serves as a model for studying DIET-mediated syntrophy (Summers et al., 2010). In this co-culture, *G. sulfurreducens* alone cannot use ethanol as an electron donor and *G. metallireducens* cannot use fumarate as an electron acceptor, but their cooperation can achieve ethanol metabolism with the reduction of fumarate (Summers et al., 2010; Smith et al., 2015). To date, conductive pili and some *c*-type cytochromes (*c*-Cyts) are considered to be the main pathways mediating DIET. Conductive pili are not only the conduit for long-distance electron transfer between cells and also play a role in establishing cell-to-cell contacts in biofilms (Summers et al., 2010; Ueki et al., 2018). Extracellular *c*-Cyts, such as Gmet_2896 cytochrome of *G. metallireducens* and OmcS of *G. sulfurreducens*, are demonstrated to be indispensable in DIET and the deletion of either of them fails to form co-cultures (Summers et al., 2010; Shrestha et al., 2013; Liu et al., 2018). Moreover, the expression level of flagella-related genes of *G. metallireducens* is higher in the co-culture and the flagella-deficient mutant cannot form co-culture with *G. sulfurreducens*, suggesting that flagella may play an important role in cell-cell aggregation like pili (Shrestha et al., 2013).

Exopolysaccharides (EPSs) are a kind of important extracellular matrix and play an active role in maintaining cell membrane structure, host immune defense, signal transduction, and biofilm formation (Cuthbertson et al., 2010; Flemming and Wingender, 2010). However, the effect of exopolysaccharides on the extracellular electron transfer (EET) of electroactive microorganisms is poorly understood. Our recent studies found that exopolysaccharides promote intercellular aggregation and early biofilm formation of *G. sulfurreducens*. More importantly, exopolysaccharides are associated with the EET process by anchoring extracellular *c*-Cyts and affecting pili expression (Zhuang et al., 2020, 2022). Considering the important functions of pili and *c*-Cyts on DIET and the fact that exopolysaccharides, pili, and *c*-Cyts are all localized in extracellular polymeric substances (EPS) and interact with each other (Leang et al., 2010; Bonanni et al., 2013; Liu et al., 2014), the role of exopolysaccharides in DIET deserves special attention.

Previous studies have identified genes involved in exopolysaccharides biosynthesis and export in *G. sulfurreducens* (Rollefson et al., 2009), but up to present have not been identified in *G. metallireducens*. Here, we constructed a co-culture of an exopolysaccharides-deficient mutant of *G. sulfurreducens* with *G. metallireducens* to study the role of exopolysaccharides in DIET. We found that exopolysaccharides are an important matrix for electron transfer and aggregate formation of the co-culture. The deficiency of exopolysaccharides affected the expression of pili and *c*-Cyts in the co-culture. Furthermore, the addition of conductive materials accelerated the metabolism of the exopolysaccharides-deficient co-culture. Our findings reveal an important role of exopolysaccharides in the DIET-mediated syntrophic processes, which provides a new perspective for understanding the mechanism of DIET.

## Materials and Methods

### Strains and Cultivation Condition

*Geobacter metallireducens* GS15 (DSM 7210) and *G. sulfurreducens* PCA (DSM 12127) were purchased from German Collection of Microorganisms and Cell Cultures (DSMZ, Braunschweig, Germany). The strain PCAΔ1501, an exopolysaccharides-deficient mutant of *G. sulfurreducens* PCA, was constructed following the method in our previous study (Zhuang et al., 2022). *G. metallireducens* strain was cultivated in an ferric citrate-acetate (FCA) medium, with 15 mM acetate as the electron donor and 55 mM Fe(III) citrate as the electron acceptor (Lovley et al., 1993). *G. sulfurreducens* strains were cultivated in NB acetate-fumarate (NBAF) medium, with 15 mM acetate as the electron donor and 40 mM fumarate as the electron acceptor (Coppi et al., 2001). The first generation of the co-cultures was initiated with a 5% inoculum of *G. metallireducens* and a 5% inoculum of *G. sulfurreducens* (wild type or mutant type) in an NB ethanol-fumarate (NBEF) medium containing 20 mM ethanol as the electron donor and 40 mM fumarate as the electron acceptor (Liu et al., 2018). When the fumarate was consumed completely, the co-cultures were routinely transferred into the fresh NBEF medium with a 5% inoculum. The metabolic rates of the co-cultures became faster with each transfer and approached stable after 10 transfers. In the following texts, the wild-type co-culture of *G. metallireducens* GS15 and *G. sulfurreducens* PCA was denoted as GS15 and PCA and the exopolysaccharides-deficient co-culture of *G. metallireducens* GS15 and G. *sulfurreducens* PCAΔ1501 was denoted as GS15 and PCAΔ1501. All the culturing was performed at 30°C under strictly anaerobic conditions with an N_2_/CO_2_ (80:20, v/v) gas phase.

### Bacterial Quantification

The primers used for bacterial quantification are given in Supplementary Table 1. After fumarate was consumed completely, the co-cultures were shaken vigorously and a 2-ml culture medium was used to extract the total genomic DNA with the TIANamp Bacteria DNA Kit (Tiangen, Beijing, China). The unique DNA fragments of *G. metallireducens* and *G. sulfurreducens* were amplified by primer pairs qGmetf/qGmetr and qGsulff/qGsulfr, respectively. The two DNA fragments were connected with pMD19-T plasmid (Takara, Kusatsu, Japan) for T-A cloning and then the plasmids were verified by primer pair M13f/M13r. The plasmids were extracted with the GeneJET Plasmid Miniprep Kit (Thermo Fisher Scientific, Waltham, MA, United States) and then gradient diluted, which was used for the standard solution of quantitative real-time PCR (qPCR). Unique DNA fragments and standard solution were simultaneously quantified with a real-time quantitative PCR detection system (CFX Connect, Bio-Rad, Hercules, CA, United States) to obtain the number of *G. metallireducens* and *G. sulfurreducens* in the co-cultures (Huang et al., 2020).

### Transcriptomic Analysis

The co-cultures were centrifuged under anaerobic conditions and the cells were collected, quick-frozen in liquid nitrogen, and stored at –80°C. The total RNA was extracted with TRIzol Reagent (Invitrogen, Carlsbad, CA, United States) and the ribosomal RNA (rRNA) transcripts in total RNA samples were reduced by ALFA-SEQ rRNA Depletion Kit (mCHIP, Guangzhou, China). RNA integrity was accurately detected using the Agilent 4200 System (Agilent Technologies, Waldbronn, Germany). Whole mRNAseq libraries were constructed by Guangdong Magigene Biotechnology Corporation Ltd. (Guangzhou, China) using the NEBNext Ultra Directional RNA Library Prep Kit for Illumina (New England Biolabs, Ipswich, MA, United States) following the manufacturer’s recommendations. The clustering of the index-coded samples was performed on a cBot Cluster Generation System. After cluster generation, the library was sequenced on an Illumina Novaseq6000 platform and 150 bp paired-end reads were generated. The raw data of fastq format were processed by Trimmomatic (version 0.36), the clean reads were mapped to the National Center for Biotechnology Information (NCBI) Rfam databases to remove the rRNA sequences by Bowtie2 (version 2.33), and then the data were used to map against the published reference genome of *G. metallireducens* GS15 (NC_007517.1) and *G. sulfurreducens* PCA (NC_002939.5). HTSeq-count (version 0.9.1) was used to obtain the read count and function information of each gene according to the result of the mapping. To make the expression level of genes to be comparable among different genes and different experiments, the fragments per kilobase of transcript per million fragments mapped (FPKM) of each gene was calculated. In our analysis, genes with the | log2(fold change) | ≥ 1 were considered to be significantly differentially expressed genes. The gene expression was statistically analyzed by ANOVA. Heat maps used to analyze gene expression were drawn by TBtools (version 0.66443) (Chen et al., 2020).

### Microscopy

A drop of the co-culture was added to the carbon-coated copper grid (mCHIP, Guangzhou, China) and the liquid was absorbed with filter paper after soaking for 5 min. The co-culture on the carbon-coated copper grid was negatively stained with 2% uranyl acetate for 45 s and then the dye was absorbed with filter paper. Finally, the morphology of aggregates and the number of pili in the samples were visualized using transmission electron microscopy (TEM) (H-7650, Hitachi, Tokyo, Japan) (Liu et al., 2018).

### Spectral Measurement of *c*-Cyts

When the electron acceptor in the culture medium was completely reduced, the EPS of the co-cultures was extracted as previously described (Yang et al., 2019). The electronic absorption spectra of *c*-Cyts in EPS were measured using UV-Vis Spectrophotometer (UV-2600, Shimadzu, Tokyo, Japan) and the intensity of the spectra were used to characterize the relative content of *c*-Cyts (Nakamura et al., 2009). The spectra of oxidized *c*-Cyts were obtained by directly scanning EPS samples exposed to air. For reduced *c*-Cyts, sodium dithionite was added to the EPS samples before scanning the reduced *c*-Cyts (Zhuang et al., 2020).

### Analytical Techniques

To monitor the metabolism of the co-cultures, the culture medium was periodically sampled and filtered with a 0.22-μm membrane filter and then the concentrations of ethanol and organic acids in the samples were determined as previously described (Liu et al., 2018). Briefly, ethanol concentration was detected using gas chromatography (456-GC, SCION, Karlsruhe, ND, United States) equipped with a headspace automatic sampler and a flame ionization detector (FID). The concentrations of acetate and succinate were detected using high-performance liquid chromatography (LC-20AT, Shimadzu, Kyoto, Japan) equipped with Inertsil ODS-SP column and Aminex HPX-87H column, respectively.

### Conductive Materials Addition Experiment

Granular activated carbon (GAC) (Sigma-Aldrich, St. Louis, MI, United States) was selected in 8–20 mesh sizes as previously reported (Liu et al., 2012). Magnetite was synthesized following a previous method (Kang et al., 1996) and verified with X-ray powder diffraction (D8 ADVANCE, Bruker, Germany), as shown in Supplementary Figure 1. When noted, GAC (2 g) and magnetite (0.1 g) were added to NBEF medium (80 ml), respectively, before autoclaving (Liu et al., 2012, 2015).

## Results and Discussion

### Exopolysaccharides Are an Important Extracellular Matrix for Syntrophic Growth

**FIGURE 1:**
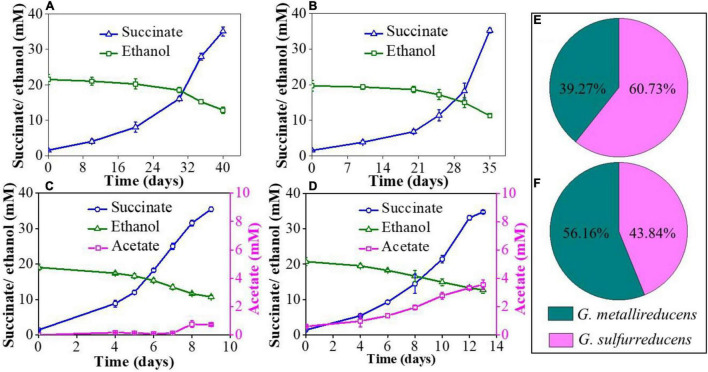
Ethanol consumption, acetate accumulation, and succinate production: **(A)** the GS15 and PCA co-culture in the first generation; **(B)** the GS15 and PCAΔ1501 co-culture in the first generation; **(C)** the GS15 and PCA co-culture in the metabolic stable period; and **(D)** the GS15 and PCAΔ1501 co-culture in the metabolic stable period. The proportion of GS15 and PCA in the co-cultures: **(E)** the GS15 and PCA co-culture in the metabolic stable period; **(F)** the GS15 and PCAΔ1501 co-culture in the metabolic stable period.

In the NBEF medium, *G. metallireducens* or *G. sulfurreducens* cannot couple ethanol oxidization and fumarate reduction alone, but the co-culture of *G. metallireducens* and *G. sulfurreducens* can achieve the complete metabolism through syntrophic cooperation (Summers et al., 2010). The metabolic kinetics of co-cultures was regularly monitored by measuring ethanol consumption, acetate accumulation, and succinate production. To eliminate the possibility that the gene GSU1501 deletion affects the metabolic characteristic of *G. sulfurreducens*, the mutant strain PCAΔ1501 was inoculated into the NBEF medium and the results showed that PCAΔ1501 could not grow in the NBEF medium (Supplementary Figure 2), which was same as the wild strain. Both the GS15 and PCA co-culture and GS15 and PCAΔ1501 co-culture had a long metabolic period for the first generation, taking 40 and 35 days to complete the metabolism, respectively (Figures 1A,B). Acetate was not detectable in the first generation of co-culture, which was due to the interference of Fe(III) citrate in the FCA medium introduced by the inoculation of *G. metallireducens*. In the following transfers, the interference disappeared by dilution and acetate became detectable. The stable metabolic periods of the GS15 and PCA co-culture and GS15 and PCAΔ1501 co-culture were much shorter than that of the first generation (Figures 1C,D), suggesting that after multiple transfers, IET between syntrophic partners became more effective (Summers et al., 2010). Notably, the metabolic period of the GS15 and PCAΔ1501 co-culture (13 days) was 44.4% longer than that of the GS15 and PCA co-culture (9 days), indicating that the deficiency of exopolysaccharides slowed the metabolism of the *Geobacter* co-culture and was not conducive to syntrophic growth.

Previous studies have shown that *G. sulfurreducens* accounts for the majority of the cells in the stable co-culture of *G. metallireducens* and *G. sulfurreducens*. For example, a ratio of 15%:85% was determined in a study by Summers et al. (2010) and a ratio of 31%:69% was reported in a study by Huang et al. (2020). The reason for this phenomenon may be that *G. sulfurreducens* utilized both the electrons and acetate produced by *G. metallireducens* oxidizing ethanol to acetate (Eqs 1 and 2) (Pravin et al., 2013):

CH_3_CH_2_OH + H_2_O → CH_3_COO^–^ + 4e^–^ + 5H^+^ (1)

CH_3_COO^–^ + 2H_2_O → 2CO_2_ + 8e^–^ + 7H^+^ (2)

In this study, the ratio of *G. metallireducens* to *G. sulfurreducens* in the GS15 and PCA co-culture was 39%:61% in the metabolic stable period (Figure 1E), which was roughly consistent with the previous observations. However, the ratio of *G. metallireducens* to *G. sulfurreducens* in the GS15 and PCAΔ1501 co-culture was 56%:44% (Figure 1F). The decreased proportion of *G. sulfurreducens* in the GS15 and PCAΔ1501 co-culture might explain the higher accumulation of acetate in the mutant co-culture than in the wild co-culture (Figures 1C,D) since *G. sulfurreducens* is the main acetate consumer in the co-culture.

### Exopolysaccharides Contribute to *c*-Cyts Anchoring in Co-cultures

**FIGURE 2:**
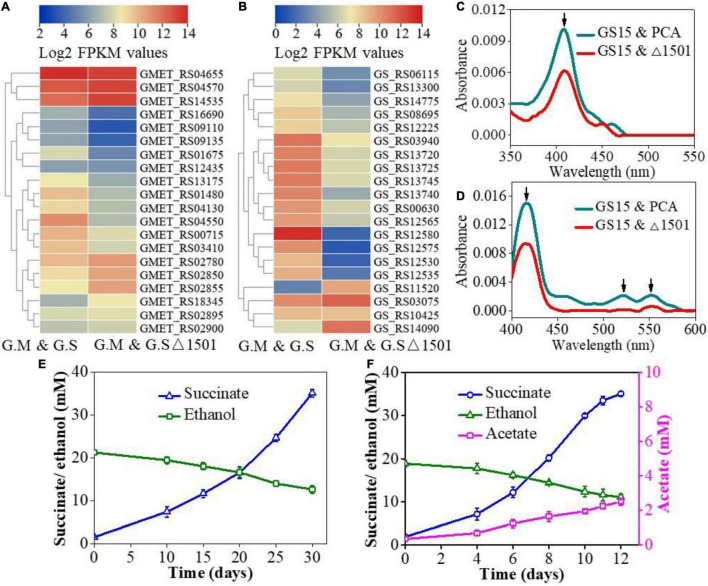
Heat map comparison of *c*-type cytochromes (*c*-Cyts)-related genes of *Geobacter metallireducens* (*G. metallireducens*) **(A)** and *Geobacter sulfurreducens* (*G. sulfurreducens*) **(B)** in the GS15 and PCA co-culture and the GS15 and PCAΔ1501 co-culture. UV-Vis spectra of oxidized *c*-Cyts **(C)** and reduced *c*-Cyts **(D)** in exopolysaccharide (EPS) from the co-cultures. Effects of magnetite addition on the syntrophic metabolism of the GS15 and PCAΔ1501 co-culture in the first generation **(E)** and metabolic stable period **(F)**.

*c*-type cytochromes (*c*-Cyts) are a kind of redox-active substances that mediate electron transfer (Estevez-Canales et al., 2015) and extracellular *c*-Cyts are essential for electrical connection in DIET (Summers et al., 2010; Shrestha et al., 2013). In this study, *c*-Cyts-related genes with significant differences (*P* < 0.05) in the expression levels of *G. metallireducens* and *G. sulfurreducens* in the co-culture of GS15 and PCA and GS15 and PCAΔ1501 are presented with a heat map comparison. For *G. metallireducens*, the deficiency of exopolysaccharides resulted in significant upregulation of 4 *c*-Cyts genes (2.1 < fold change < 4.0) and significant downregulation of 10 *c*-Cyts genes (3.9 < fold change < 18.1) (Figure 2A). For *G. sulfurreducens*, the deficiency of exopolysaccharides resulted in significant upregulation of 3 *c*-Cyts genes (2.4 < fold change < 109.8) and significant downregulation of 16 *c*-Cyts genes (9.6 < fold change < 4377.2) (Figure 2B). Transcription of *c*-Cyts genes such as OmcE (GS_RS03075) increased by 2.4-fold and OmcZ (GS_RS10425), OmcF (GS_RS12225), OmcS (GS_RS12580), OmcC (GS_RS13720), and OmcB (GS_RS13740) decreased by 1. 1-, 5. 6-, 4377. 2-, 19. 6-, and 62.7-fold, respectively. Among these extracellular *c*-Cyts, Gmet_2896 cytochrome (GMET_RS14535) of *G. metallireducens* and OmcS (GS_RS12580) of *G. sulfurreducens* have been demonstrated to play a key role in DIET and the deletion of Gmet_2896 cytochrome or OmcS would fail the co-culture metabolizing ethanol and fumarate (Summers et al., 2010; Liu et al., 2018). The expression level of Gmet_2896 cytochrome gene was upregulated by 2.3-fold and *OmcS* gene was downregulated by 4377.2-fold in this study (Figures 2A,B). The significant decrease of OmcS expression in the GS15 and PCAΔ1501 co-culture might be the main cause for its sluggish metabolism compared with the wild-type co-culture.

Considering the importance of extracellular *c*-Cyts in DIET (Shi et al., 2009), the extracellular *c*-Cyts in EPS from the two co-cultures were determined. UV–Vis spectra of the oxidized *c*-Cyts (Figure 2C) and the reduced *c*-Cyts (Figure 2D) showed obvious characteristic peaks of *c*-Cyts, which were consistent with previous reports (Heitmann and Einsle, 2005). Since the absorbance of the characteristic peaks is directly proportional to the concentration of *c*-Cyts, the relative content of extracellular *c*-Cyts of the GS15 and PCAΔ1501 co-culture was lower than that of the GS15 and PCA co-culture (Figures 2C,D). Since exopolysaccharides have the function of anchoring extracellular *c*-Cyts (Rollefson et al., 2011), the decrease of exopolysaccharides consequently resulted in less accumulation of extracellular *c*-Cyts in the EPS of the co-culture.

Previous studies found that the addition of magnetite to the co-culture of *G. metallireducens* and *G. sulfurreducens* significantly reduced the transcription level of *OmcS* gene and the addition of magnetite to the OmcS-deficient co-culture would stimulate the metabolic kinetics to the level same as that of the wild-type co-culture (Liu et al., 2015). Magnetite, thus, is proposed to be able to compensate for the electron transfer function of OmcS between *Geobacter* species and is widely used to facilitate DIET-mediated syntrophic interactions (Kato et al., 2012; Tang et al., 2016). Based on the decrease of *c*-Cyts and dramatic reduction of OmcS expression in the GS15 and PCAΔ1501 co-culture (Figures 2B–D), magnetite was added to explore their compensatory role in the growth and metabolism of the exopolysaccharides-deficient co-culture. The results of abiotic control showed that ethanol remained unchanged and no acetate and succinate were detected when magnetite was added to the NBEF medium (Supplementary Figure 3), indicating that chemical reactions were not involved between magnetite and the components in the NBEF medium. For the first generation of the GS15 and PCAΔ1501 co-culture, the amendment of magnetite shortened the metabolic period by 14.3% (Figures 1B, 2E), while for the metabolic stable co-culture of GS15 and PCAΔ1501, the addition of magnetite faster metabolic period by 7.7% compared with the co-culture without magnetite (Figures 1D, 2F). These results indicated that the stimulatory effect of magnetite on DIET metabolism of the GS15 and PCAΔ1501 co-culture was more evident for the first generation than in the metabolic stable period. These results suggested that with sufficient time for adaption, the GS15 and PCAΔ1501 co-culture were developing the effective biological electrical connections that became comparable to magnetite.

### Exopolysaccharides Contribute to Aggregates Formation of Co-cultures

**FIGURE 3:**
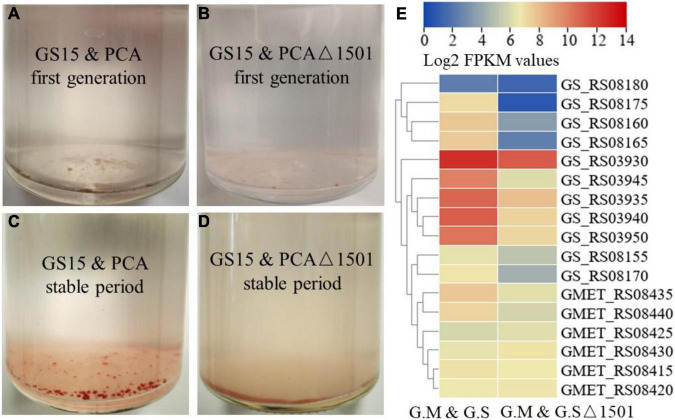
Morphology of the GS15 and PCA co-culture **(A)** and the GS15 and PCAΔ1501 co-culture **(B)** in the first generation. Morphology of the GS15 and PCA co-culture **(C)** and the GS15 and PCAΔ1501 co-culture **(D)** in the metabolic stable period. Heat map comparison of related genes of hybrid subunits and flavin of *G. metallireducens* and *G. sulfurreducens* in the GS15 and PCA co-culture and the GS15 and PCAΔ1501 co-culture **(E)**.

The co-culture of *G. metallireducens* and *G. sulfurreducens* will form conductive aggregates and the conductive aggregates contribute to the close contact between syntrophic partners, thereby shortening electron transfer distance and reducing energy loss (Summers et al., 2010; Lovley, 2017). Summers et al. (2010) found that the GS15 and PCA co-culture favored forming of large, tight spherical aggregates with sequential transfers and the larger aggregates corresponded faster metabolic rate. In the first generation, both the GS15 and PCA co-culture and the GS15 and PCAΔ1501 co-culture formed very small flocks (Figures 3A,B). After approaching the metabolic stable period, the GS15 and PCA co-culture formed reddish, large, and tight spherical aggregates, which were similar to previously observed after multiple transfers of the co-culture (Summers et al., 2010; Liu et al., 2018), while the GS15 and PCAΔ1501 co-culture failed to form similar aggregates and existed in loose flock-like structure (Figures 3C,D). As one of the main components in EPS, exopolysaccharides are highly associated with microbial aggregates of aerobic granules (Tay et al., 2001; Adav et al., 2008). Our results first demonstrated the important role of exopolysaccharides in the formation and stability of DIET aggregates.

In the co-culture of *G. metallireducens* and *G. sulfurreducens*, hydrogen-mediated MIET is not definitively excluded. Membrane-bound hybrid is the only hydrogenase for *G. sulfurreducens*, the transcription levels of all the genes encoding hybrid subunits (GS_RS03930 to GS_RS03950) of *G. sulfurreducens* were significantly reduced (> 2.7-fold) in the GS15 and PCAΔ1501 co-culture compared to those in the wild co-culture (Figure 3E). Recent studies have shown that *Geobacter* could secrete flavins with redox activity and flavins could mediate IET in the co-culture of *G. metallireducens* and *G. sulfurreducens* (Huang et al., 2020). In this study, the transcription levels of the flavin synthesis genes of both the *G. metallireducens* (GMET_RS08435 and GMET_RS08440) and *G. sulfurreducens* (GS_RS08155 to GS_RS08180) were significantly reduced (> 2.8- and > 2.5-fold, respectively) in the GS15 and PCAΔ1501 co-culture relative to the wild co-culture (Figure 3E). In sum, the deficiency of exopolysaccharides in the co-culture impaired the MIET-mediated syntrophic metabolism through hydrogen and flavin, since close interaction of syntrophic microorganisms is also necessary for efficient MIET.

**FIGURE 4:**
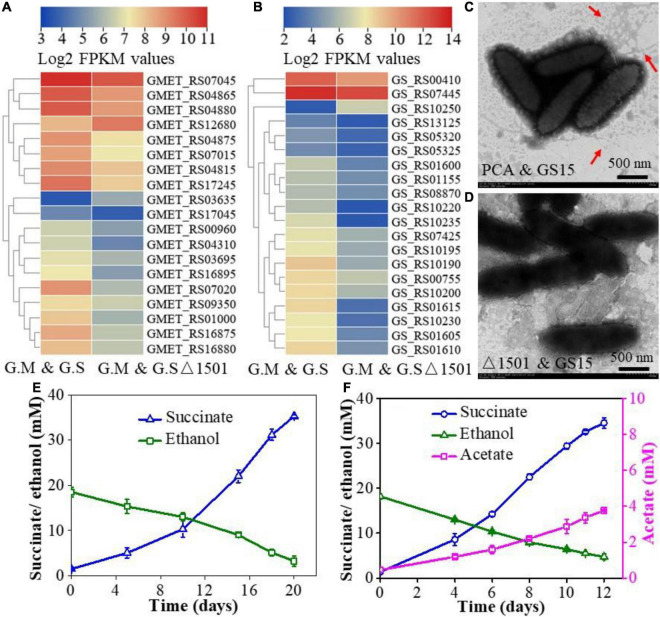
Heat map comparison of pili-related genes of *G. metallireducens* **(A)** and *G. sulfurreducens* **(B)** in the GS15 and PCA co-culture and the GS15 and PCAΔ1501 co-culture. Transmission electron microscopy (TEM) images of the GS15 and PCA co-culture **(C)** and GS15 and PCAΔ1501 co-culture **(D)**. Effects of granular activated carbon (GAC) addition on the syntrophic metabolism of the GS15 and PCAΔ1501 co-culture in the first generation **(E)** and metabolic stable period **(F)**.

The syntrophic growth of *G. metallireducens* and *G. sulfurreducens* requires the participation of pili and deletion of either pili of *G. metallireducens* or *G. sulfurreducens* leads to the failure of the co-culture to perform syntrophic metabolism (Summers et al., 2010; Shrestha et al., 2013). To gain insight into the difference in pili expression between the wild-type co-culture and the exopolysaccharides-deficient co-culture, the transcriptional abundances of pili-related genes in the two co-cultures were compared. The results showed that the expression levels of pili-related genes of both the *G. metallireducens* and *G. sulfurreducens* were lower in the GS15 and PCAΔ1501 co-culture than in the GS15 and PCA co-culture (Figures 4A,B). The morphology of aggregates and the number of pili were observed under transmission electron microscopy. In the GS15 and PCA co-culture, these microorganisms were closely clustered and the pili were abundant, while in the GS15 and PCAΔ1501 co-culture, these microorganisms were relatively dispersed and it was difficult to observe pili (Figures 4C,D). These observations were consistent with the co-culture morphology (Figure 3D) and the transcription level of pili-related genes (Figures 4A,B). A dense pili network is commonly observed in the co-cultures of *Geobacter* species (Lovley, 2017), in which pili not only help syntrophic partners to form aggregates but also facilitate long-distance electron transfer (Reguera et al., 2005; Summers et al., 2010).

Granular activated carbon is a kind of carbon material with a dense microporous structure, large surface area, and high electrical conductivity (Liu et al., 2012). Previous studies have found that the addition of GAC was able to restore the growth of pili-deficient co-culture (Liu et al., 2012; Lovley, 2017). Here, we try to compensate for the decrease of pili in the GS15 and PCAΔ1501 co-culture by adding GAC. The results of the abiotic control showed that after adding GAC to NBEF medium for 30 days, the ethanol concentration decreased by 38.4% and acetate and succinate were not detected (Supplementary Figure 4), indicating that GAC only physically adsorbed ethanol. For the first generation of the GS15 and PCAΔ1501 co-culture, the amendment of GAC shortened the metabolic period by 42.9% (Figures 1B, 4E), while for the metabolic stable co-culture of GS15 and PCAΔ1501, the addition of GAC fasters the metabolic period only by 7.7% (Figures 1D, 4F). These results indicated that the stimulatory effect of GAC on DIET metabolism of the GS15 and PCAΔ1501 co-culture was more evident for the first generation than the metabolic stable period, which was the same as the results of adding magnetite. According to a previous study, in the presence of GAC, cells in syntrophic co-cultures were not closely aggregated but attached to GAC, which proposed that GAC can provide sufficient interspecies electrical connection and relieve the necessity for close physical connection for syntrophic partners (Liu et al., 2012). Therefore, our results indicated that both the magnetite and GAC had a very limited stimulatory effect on the metabolism of the adapted GS15 and PCAΔ1501 co-culture, which implied that electrons might be transferred *via* another mechanism that was not highly associated with the compensatory role of magnetite or the GAC.

## Conclusion

The process of DIET is considered to depend on conductive components such as pili and *c*-Cyts. In this study, we demonstrated that non-conductive exopolysaccharides are also an important matrix in DIET. The deficiency of exopolysaccharides prevented typical aggregate formation in the co-culture of *G. metallireducens* and *G. sulfurreducens* and slowed the metabolic rate of co-culture. Furthermore, the deficiency of exopolysaccharides decreased the expression of both the pili and *c*-Cyts, which was not conducive to direct electron transfer between syntrophic partners. Magnetite and GAC were added, respectively, to compensate for the decrease of *c*-Cyts and pili and the results showed that the addition of conductive materials could effectively promote the metabolic rate of co-culture in the first generation, but the facilitated effect on the metabolic stable period co-culture was limited. The roles of exopolysaccharides were proposed as an indispensable component for DIET co-culture to develop typical aggregate and an important matrix for anchoring electrical linkage between syntrophic cells. These findings enrich the mechanism of DIET and provide a new perspective for related studies.

## Data Availability Statement

The raw data supporting the conclusions of this article will be made available by the authors, without undue reservation.

## Author Contributions

LZ, GY, and ZZ designed the experiments. ZZ and XX carried out the experiments. ZZ wrote the initial version of the manuscript. LZ and GY contributed to the critical review of the manuscript. All authors have read, commented, and approved the final version of the manuscript.

## Conflict of Interest

The authors declare that the research was conducted in the absence of any commercial or financial relationships that could be construed as a potential conflict of interest.

## Publisher’s Note

All claims expressed in this article are solely those of the authors and do not necessarily represent those of their affiliated organizations, or those of the publisher, the editors and the reviewers. Any product that may be evaluated in this article, or claim that may be made by its manufacturer, is not guaranteed or endorsed by the publisher.
