# Supplementary material for: The Role of Exopolysaccharides in Direct Interspecies Electron Transfer
Source: Front Microbiol. 2022 Jun 16;13:927246. doi: 10.3389/fmicb.2022.927246 (PMC9244359; doi:10.3389/fmicb.2022.927246)
Supplement: Supplementary file 1 [file Data_Sheet_1.docx]

***Supplementary Material***

**The Role of Exopolysaccharides in** **Direct Interspecies Electron Transfer**

*Zheng Zhuang, Xue Xia, Guiqin Yang^*^, Li Zhuang^*^*

*Guangdong Key Laboratory of Environmental Pollution and Health, School of Environment, Jinan University, Guangzhou 510632, China*

*Corresponding Author:

Guiqin Yang, E-mail: gqyang@jnu.edu.cn

Li Zhuang, E-mail: zhuangli@jnu.edu.cn

Supplementary Table 1. Primers used in this study

| Primer name | Sequence (5’ to 3’) | Purpose |
| --- | --- | --- |
| qGmetf | ATGGCCCACATCTTCATCTC | qPCR |
| qGmetr | TGCATGTTTTCATCCACGAT |  |
| qGsulff | CCAGCTACGCCTACTTCTTCTTT | qPCR |
| qGsulfr | AAGCTGTGGTTCAGGAGGTATTT |  |
| M13f | TGTAAAACGACGGCCAGT | Verification |
| M13r | CAGGAAACAGCTATGACC |  |


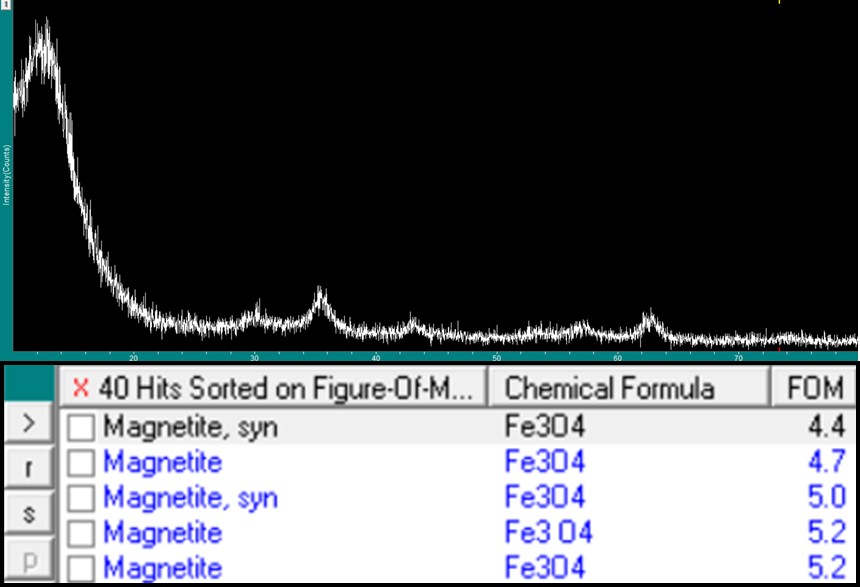


Supplementary Figure 1. XRD pattern of magnetite synthesized in the lab.

Supplementary Figure 2. Ethanol consumption, acetate accumulation and succinate production in the NBEF medium inoculated with the mutant PCA△1501 only.

Supplementary Figure 3. Ethanol consumption, acetate accumulation and succinate production in the NBEF medium supplemented with magnetite only.

Supplementary Figure 4. Ethanol consumption, acetate accumulation and succinate production in the NBEF medium supplemented with GAC only.
